# Vitamin B12 Intake From Animal Foods, Biomarkers, and Health Aspects

**DOI:** 10.3389/fnut.2019.00093

**Published:** 2019-06-28

**Authors:** Rima Obeid, Sandra G. Heil, Maxime M. A. Verhoeven, Ellen G. H. M. van den Heuvel, Lisette C. P. G. M. de Groot, Simone J. P. M. Eussen

**Affiliations:** ^1^Department of Clinical Chemistry and Laboratory Medicine, Saarland University Hospital, Homburg, Germany; ^2^Department of Clinical Chemistry, Erasmus MC University Medical Center Rotterdam, Rotterdam, Netherlands; ^3^Department of Rheumatology and Clinical Immunology, UMC Utrecht, Utrecht, Netherlands; ^4^FrieslandCampina, Amersfoort, Netherlands; ^5^Division of Human Nutrition, Wageningen University, Wageningen, Netherlands; ^6^Department of Epidemiology, CARIM School for Cardiovascular Diseases Maastricht University, Maastricht, Netherlands

**Keywords:** vitamin B12 (cobalamin), intake, animal food products, health, infants, pregnancy, elderly

## Abstract

The EAT-Lancet commission recently suggested that transformation to healthy diets by 2050 will require a reduction of at least 50% in consumption of foods such as red meat and sugar, and a doubling in the global consumption of fruits, vegetables, nuts, and legumes. A diet rich in plant-based foods and with fewer animal source foods confers both improved health and environmental benefits. Notably, the risk of vitamin B12 deficiency increases when consuming a diet low in animal products. Humans are dependent on animal foods such as dairy products, meat, fish and eggs. Vitamin B12 deficiency is common worldwide, especially in populations with low consumption of animal foods because of low socioeconomic status, ethical reasons, or because of their lifestyle (i.e., vegans). According to the European Food Safety Authoroty, the recommended adequate intake of vitamin B12 is 4.0 μg/d for adults, and vitamin B12 requirements are higher during pregnancy and lactation. Infants and children from deficient mothers and elderly people are at risk for vitamin B12 deficiency. Diagnosis of vitamin B12 deficiency is hampered by low specificity of available biomarkers, and there is no consensus yet regarding the optimal definition of low vitamin B12 status. In general, a combination of at least two biomarkers is recommended. Therefore, this review presents an overview of vitamin B12 biochemistry and its biomarkers. We further summarize current recommendations of vitamin B12 intake, and evidence on the associations of vitamin B12 intake from different nutrient-dense animal foods with vitamin B12 status markers. Finally, potential consequences of low vitamin B12 status on different health outcomes for pregnant women, infants and elderly are presented.

## Vitamin B12

### Food Sources of Vitamin B12

Vitamin B12 (cobalamin) is an essential water-soluble micronutrient of microbial origin (1). It is naturally found in animal food products, including meat, poultry, (shell)fish, eggs, milk, and other dairy products (2). Vitamin B12 is generally not present in plant foods, but fortified breakfast cereals are a readily available source of vitamin B12 with high bioavailability (3, 4). Some nutritional yeast products also contain vitamin B12. This paper will only focus on vitamin B12 intake from natural food products, e.g. animal foods. According to recent results of the 2012-2016 Dutch Food Consumption Survey, the contribution of dairy, meat, (shell) fish, supplements, and eggs to total vitamin B12 intake is 38.5, 30, 8.5, 8.4, and 4.6%, respectively^1^. The nutrient dense animal food products rich in vitamin B12 are also rich in other nutrients such as zinc, iron, vitamin D, and proteins. To the best of our knowledge, interactions of vitamin B12 with these nutrients are not fully established, and it is not unlikely that associations of low vitamin B12 with health outcomes may be modified by deficiency of these nutrients or the presence of disorders that affect the digestive system. Notably, vitamin B12 and folate act together within the one-carbon metabolism, and paragraph 4.3.1 will elaborate on a potential role of high folate and low vitamin B12 status in health.

### Uptake of Vitamin B12

Vitamin B12 plays an important role in one-carbon metabolism. Dietary vitamin B12 is, once ingested, bound to haptocorin (an animal protein), which carries vitamin B12 to the stomach. In the stomach, HCl and pepsin are released which release vitamin B12 from animal proteins. Free vitamin B12 then binds to haptocorrin in the stomach after which it is transported into the intestine, where vitamin B12 is released by pancreatic enzymes after which vitamin B12 binds to intrinsic factor (IF) (5). Vitamin B12 -IF complex binds to the cubulin receptor in the distal ileum, which takes up vitamin B12 through receptor-mediated endocytosis (5). Once taken up, vitamin B12 is released to the plasma where it is bound to its transport proteins; haptocorrin (HC) and trancobalamin (TC) (6, 7). In the circulation, 20–25% of vitamin B12 is bound to TC (called holo-TC or active B12), which is taken up and used by the cells. The other 75–80% of vitamin B12 is bound to HC, which is stored in the liver (6–8).

### Causes of Acquired Vitamin B12 Deficiency

Vitamin B12 deficiency increased with age and is mostly due to malabsorption of the vitamin. In addition, low intake of animal food products—as outlined in chapter 3–and use of certain drugs may also result in vitamin B12 deficiency (Table 1). Absorption of vitamin B12 is dependent upon several processes including IF production. If gastric IF production is impaired, like when gastric parietal cells are destructed in case of gastritis or when less gastric parietal cells are strongly reduced in case of a gastric bypass less, this will result in reduced absorption of vitamin B12. Uptake of vitamin B12 takes place in the distal ileum, and in case of an ileal dissection, bacterial overgrowth or intestinal diseases such as Crohn's disease less vitamin B12 can be taken up by the ileal cells resulting in lower intake of vitamin B12. Drugs that regulate secretion of gastric acid production such as proton-pomp inhibitors can also lead to vitamin B12 deficiency due to in impaired release of vitamin B12 from food proteins. In addition, metformin, a drug that is used to lower glucose levels, has been shown to results in lower vitamin B12 levels in serum most likely due to interfering with calcium-related binding of IF-B12 complex to the cubulin receptor (9). Considering drugs the party drug nitrous oxide has gained a lot of attention recently as it has been shown that high intake of nitrous oxide can result in vitamin B12 deficiency due to irreversible oxidation of the cobalt ion of MeCbl and AdoCbl, which makes both coenzymes inactive resulting in increased levels of methylmalonic acid (MMA) and homocysteine (10). Vitamin B12 and active B12 levels are mostly not low in serum in case of nitrous oxide overabuse and functional markers such as MMA and homocysteine should be used for laboratory diagnosis (10).

**Table 1:** Causes of acquired vitamin B12 deficiency.

**Cause**	**Effect**
**MALABSORPTION**	
Gastric bypass	↓ IF production
Gastrointestinal infection with H. Pylori	↓ IF production
Ileal resection	↓ Absorption of B12-IF
Bacterial overgrowth	↓ Absorption of B12-IF
Intestinal disease (e.g., Crohn)	↓ Absorption of B12-IF
Pernicious anemia	Antibodies against IF or parital cells
Difficulties in chewing foods	Releasing of B12 from food proteins
**NUTRITIONAL**	
Malnutrition	↓ Vitamin B12 consumption
Vegetarian or vegan diet	↓ Intake of B12 containing animal products
**DRUGS**	
Proton-pump inhibitors	Defective release of B12 from food
Metformin	↓ Absorption of B12
Nitrous oxide	Inactivation of methionine synthase (in case of NO)

*IF, Intrinsic factor; ↓, decreased*.

### Biochemistry of Vitamin B12

Different derivatives of cobalamin exist of which methylcobalamin (MeCbl) and adenosylcobalamin (AdoCbl) are the physiological co-enzyme forms. MeCbl is a cofactor in the methionine-synthase dependent remethylation of homocysteine into methionine, which takes place into the cytosol. This remethylation reaction is an important step of the one-carbon metabolism, in which also reduction of folate derivatives takes place, which are important for DNA synthesis. In addition, methionine is an essential amino acid which is involved in formation of the universal methyl donor S-adenosylmethionine. Low dietary intake of vitamin B12 results in elevated homocysteine levels and might affect DNA synthesis and DNA methylation. AdoCbl is involved in the l-methylmalonyl-CoA-mutase-dependent conversion of methylmalonyl-CoA into succinyl-CoA, which takes place in the mitochondrium (11). Low dietary intake of vitamin B12 results in accumulation of methylmalonyl-CoA that converts to MMA. Increasing levels of MMA are observed in plasma in case of vitamin B12 deficiency.

### Biomarkers of Vitamin B12

Several biomarkers (Table 2) exist to evaluate vitamin B12 status in blood. The most used biomarker is total vitamin B12, which measures vitamin B12 bound to both transport proteins (HC and TC), which gives a generally estimation of the vitamin B12 status in the blood (7, 11). In addition, holoTC (active B12), which is the transcobalamin-bound vitamin B12 has been suggested to be an early marker of vitamin B12 status. HoloTC can be used as an initial test to measure vitamin B12 status in blood (12–14). Both total vitamin B12 and holoTC are applied in laboratory diagnostics and functional tests such as homocysteine and MMA are used to confirm diagnosis in case of low-normal vitamin B12 status. No consensus exists about which cut-off values should be applied and which is the best marker or combination of markers to assess vitamin B12 status (15). In general, reference intervals are used or alternative cut-off values are chose based upon sensitivity and specificity (14). However, these cut-off values are not generally applicable, as total vitamin B12 and active vitamin B12 tests are not harmonized, which hampers interpretation difficult for general practitioners.

**Table 2:** Biomarkers of vitamin B12 status in serum or plasma.

**Biomarker**	**Indication**	**Interpretation**
Total vitamin B12	Global vitamin B12 status	↓ In vitamin B12 deficiency ↑ Myeloid cell proliferation ↓ Pregnancy
Holo-transcobalamin	Vitamin B12-bound to transcobalamin or active B12	↓ In vitamin B12 deficiency ↓ In TC deficiency
Methylmalonic acid	Functional marker of vitamin B12 deficiency	↑ Vitamin B12 deficiency ↑ Renal dysfunction
Homocysteine	Functional marker of vitamin B12 deficiency	↑ Vitamin B12 deficiency ↑ Renal dysfunction ↑ Folate deficiency

*↓, decreased; ↑, increased*.

### Consideration

Regarding biomarkers of vitamin B12 there is a need to establish reference intervals of total vitamin B12 in pregnancy as these levels decrease during pregnancy. In addition, better biomarkers are necessary to determin vitamin B12 deficiency as total B12 and active B12 hamper diagnostic specificity.

## Present Recommended Dietary Intake of Vitamin b12 and Their Limitations

### Dietary Reference Values for Vitamin B12

Several organizations have followed different approaches to set the dietary reference values for vitamin B12 (Table 3). The dietary reference values for adult men and women aged >18 years range between 2 and 4 μg/d depending on the judgments used. Generally, the increased requirements for vitamin B12 in women during pregnancy and lactation have been acknowledged and translated into higher reference values compared with non-pregnant women. No special intake recommendations exist for elderly people, despite the evidence that vitamin B12 malabsorption and deficiency are common in the elderly. Furthermore, the intake recommendations for infants were mainly based on outdated observational studies and on vitamin B12 content in human breastmilk. Measurement of vitamin B12 in breastmilk has been hampered by methodological problems due to the high milk haptocorrin that interferes with most available assays. In general, the European Food Safety Authority (EFSA) panel defined the Adequate Intake of vitamin B12 based on three indicators of vitamin B12 requirements (16):

1. Maintenance of heamatological markers in patients with pernicious anemia in a remission phase (i.e., correcting hemoglobin, mean corpuscular volume, and reticulocyte).
2. Maintenance of the total body stores of vitamin B12 (≈2–3 mg) by adjusting the daily requirements for the daily loss of the vitamin. The absorption efficacy of vitamin B12 from foods is assumed to be 40% and the daily loss is between 2 and 6 μg/d (biliary loss or transfer to the fetus or infant (via the placenta or the breastmilk).
3. Maintenance of normal serum levels of vitamin B12 markers (total vitamin B12, MMA, holoTC, and Hcy).

**Table 3:** Dietary reference values for vitamin B12 (in μg/d) in different age and sex groups as suggested by different organizations.

	**EFSA 2015**	**D-A-CH 2015**	**NCM 2014**	**WHO 2004**	**NL 2003**	**IOM 1998**	**SCF 1993**	**COMA 1991**
**Infants and children**								
Age	7Mo−6y	4–12Mo	6–11Mo	7–12Mo	6–11Mo	7–12Mo	6–11Mo	7–12 Mo
Reference value	1.5 μg/d	0.8 μg/d	0.5 μg/d	0.7 μg/d	0.5 μg/d	0.5 μg/d	0.5 μg/d	0.4 μg/d
Age		1–4 y	1–2 y	1–3 y	1–3 y	1–3 y	1–3 y	1–3 y
Reference value		1.0 μg/d	0.6 μg/d	0.9 μg/d	0.7 μg/d	0.9 μg/d	0.7 μg/d	0.5 μg/d
Age		4–7 y	2–5 y	4–6 y	4–8 y	4–8 y	4–6 y	4–6 y
Reference value		4.5 μg/d	0.8 μg/d	1.2 μg/d	1.3 μg/d	1.2 μg/d	0.9 μg/d	0.8 μg/d
Age	7–10 y	7–10 y	6–9 y	7–9 y	9–13 y	9–13 y	7–10 y	7–10 y
Reference value	2.5 μg/d	1.8 μg/d	1.3 μg/d	1.8 μg/d	2.0 μg/d	1.8 μg/d	1.0 μg/d	1.0 μg/d
Age	11–14 y	10–13 y	10–17 y	10–18 y	10–18 y	14–18 y	11–14 y	11–14 y
Reference value	3.5 μg/d	2.0 μg/d	2.0 μg/d	2.4 μg/d	2.8 μg/d	2.4 μg/d	1.3 μg/d	0.2 μg/d
Age	15–17 y	13–19 y					15–17 y	15–18 y
Reference value	4.0 μg/d	3.0 μg/d					1.4 μg/d	1.5 μg/d
Adults (M+F), > 18 y	4.0 μg/d	3.0 μg/d	2.0 μg/d	2.4 μg/d	2.8 μg/d	2.4 μg/d	1.4 μg/d	1.5 μg/d
Pregnant women	4.5 μg/d	3.5 μg/d	2.0 μg/d	2.6 μg/d	3.2 μg/d	2.6 μg/d	1.6 μg/d	1.5 μg/d
Lactating women	5.0 μg/d	4.0 μg/d	2.6 μg/d	2.8 μg/d	3.8 μg/d	2.8 μg/d	1.9 μg/d	2.0 μg/d

*EFSA, European Food Safety Authority; NCM, Nordic Council of Ministers; WHO, World Health Organization; NL, Health Council of the Netherlands; IOM, U.S. Institute of Medicine; SCF, Scientific Committee on Food; COMA, Committee on Medical Aspects of Food Policy*.

### Vitamin B12 Intake Necessary for Maintenance of Normal Vitamin B12 Biomarkers

Approximately 1% of a high oral vitamin B12 dose (i.e., derived from supplemental cyanocobalamin) crosses the intestinal barrier into the blood via simple diffusion (17), after saturation of IF (max. 5 μg/meal is absorbed via IF) (18). Vitamin B12 intake shows a dose-response relationship with blood vitamin B12 markers. We focused here on studies on vitamin B12 biomarkers in relation to vitamin B12 intake in the range below 50 μg/d. Studies using therapeutic doses of vitamin B12 are beyond the scope of this review.

In a study among 98 Danish post-menopausal women Bor et al. (19)suggested that an intake of 6 μg/d vitamin B12 (determined from 7-d weighed food records) is sufficient to maintain highest concentrations of vitamin B12 and holoTC, and lowest concentrations of MMA and Hcy with median (25–75th percentiles) of 380 (270–480) pmol/L for vitamin B12, 119 (92–162) pmol/L for holoTC, 0.12 (0.14–0.17) μmol/L for MMA, and 9.8 (8.3–11.4) μmol/L for Hcy compared to intakes lower than 6 μg/d. A similar study in 299 healthy US adults found that mean levels of vitamin B12 and holoTC were highest in the intake range between 4.2 and 7.0 μg/d, while plasma MMA and Hcy reached lowest levels in subjects who achieved an intake of ≥7.0 μg/d (20).

The association between vitamin B12 markers and intake was generally weaker in studies in elderly people (21) compared to those in younger people, in studies considering only dietary intake compared to studies on vitamin B12 intake from diet plus supplements, and in studies considering vitamin B12 intake up to 100 μg/d than those using larger doses (21). This could be due to a better absorption of free vitamin B12 from supplements compared to protein-bound vitamin B12 from foods. Van Asselt et al., reported a median vitamin B12 intake (from diet plus supplements) of 6.3 μg/d in elderly Dutch people (mean age 76 years) with normal vitamin B12 markers (vitamin B12 >260 pmol/L and MMA <320 nmol/L) (22). Subjects with mild vitamin B12 deficiency (vitamin B12 <260 pmol/L and MMA>320 nmol/L) had a median intake of 4.9 μg/d, and those with a possible deficiency (either low B12 or elevated MMA) had a median intake of 5.1 μg/d (22). The deficiency in elderly people could be better explained by malabsorption disorders instead of by minor variations in intakes (22). In line with this, associations of plasma concentrations of vitamin B12, MMA, and Hcy with vitamin B12 intake was not present in some studies (23, 24) possibly due to age- and disease- related malabsorption.

A meta-analysis on the association between vitamin B12 intake and biomarkers Dullemeijer et al. estimated that doubling the intake of vitamin B12 is associated with 11.0% (95% CI: 9.4%, 12.5%) higher serum vitamin B12 concentration (21). The association between vitamin B12 intake and biomarkers was stronger in studies conducted in elderly people than in adult populations, which could be related to low baseline concentrations of vitamin B12 in the elderly (21). The slope of the change of plasma vitamin B12 in relation to vitamin B12 intake flattened when vitamin B12 intake was >100 μg/d (21), which could reflect the limited proportional absorption of vitamin B12 from high dose supplements. Compared to plasma vitamin B12, the changes of serum MMA (mean −7%; 95% CI = −10 to −4%) in response to doubling vitamin B12 intake were smaller (21), which could be due to the short observational time of most studies and to the influence of renal function on MMA levels.

The increase in plasma vitamin B12 and the decrease in functional markers appear to depend on population characteristics (mainly age and accompanying diseases), duration of the intervention, starting plasma concentrations of the vitamin, and the administered dose of crystalized cyanocobalamin, even in non-therapeutic ranges. In general, a daily intake of free cyanocobalamin as low as 1.5–2.5 μg provided for approximately 4–6 months may increase plasma vitamin B12 by 50–100 pmol/L.

### Consideration

A total intake of vitamin B12 from the diet between 4 and 7 μg/d is associated with normal plasma vitamin B12 and MMA and thus appears to be adequate to maintain body vitamin B12 status in adults. This intake might be insufficient if people have difficulties in chewing foods, releasing the vitamin from its food binding, and/or absorbing it due to disorders as shown in Table 1 (25, 26). Elderly people with *H-pylori* infection (26), or food-cobalamin malabsorption (25, 27, 28) may be at risk for vitamin B12 deficiency despite sufficient dietary intake. It is unclear if elderly people would generally benefit from higher vitamin B12 intake recommendations.

## The Association of Animal Food Products Containing Vitamin b12 With Circulating Vitamin B12 Biomarkers From Observational Studies

In addition to supplements or fortified cereals as potential sources of vitamin B12, this paper focusses on vitamin B12 intake from natural food products, e.g., animal foods. In total, 19 observational studies were identified addressing associations of vitamin B12 containing aminal food items with plasma or serum vitamin B12 biomarkers. These studies were performed among infants (*n* = 1 study) (29), children (*n* = 5 studies) (30–35), pregnant women (*n* = 1 study) (36), adults (*n* = 7 studies) (3, 19, 20, 37–45), and elderly (*n* = 5 studies) (22–24, 43, 46–49). The majority of these studies had a cross-sectional design, except for some case-control study conducted among infants (29), children (30), and elderly (24), and a prospective study (3). The observational studies were heterogenous with respect to dietary assessment of animal food items or dietary patterns, usage of different vitamin B12 biomarkers, and statistical analyses, which hampers the direct comparison between studies (Table 4). Therefore, this section summarizes main findings from individual studies by different age categories.

**Table 4:** Main characteristics and results of observational studies addressing the relation between dietary intake and vitamin B12 status biomarkers among different age categories.

**Author, year, country, design**	**Population (*% female)***	**Age [range] (years)**	**Dietary intake**	**Vitamin B12 status biomarker**	**Results**	**Remarks**	**Final conclusion**
**INFANTS**							
Dagnelie et al. (29), 1989, The Netherlands, Case–control	*N* = 103	10–20 months	Macrobiotic diet (*n =* 47), omnivorous diet (*n =* 56)	vitamin B12 (pmol/L)	Geometric mean ± coefficient of variation in: Marcobiotic group: 149 ± 21.6 Control group: 404 ± 15.6 P for difference < 0.001	None of the macrobiotic fed children had ever received animal produces or vitamin B12 supplements, except for 10 out of 47 infants who had received small amounts of dairy products at some time in their live.	Plasma B12 among macrobiotic fed infants significantly lower than among the control group
**CHILDREN**							
Van Dusseldorp et al. (30), 1999, The Netherlands, Nested case–control	*N =* 167 (54%) macrobiotic diet (*n =* 73), control (*n =* 94)	12 [9–15]	List of 6 food groups, including intake of cheese, pasteurized milk, buttermilk and yogurt.	Vitamin B12 (pmol/L) MMA (μmol/L) Hcy (μmol/L)	Correlation coefficients (*P*-value) of cobalamin with: Number of years having followed a macrobiotic diet: −0.22 (*P* = 0.06, *n* = 73) Frequency of meat consumption: 0.53 (*P* < 0.0001) Frequency of chicken consumption: 0.47 (*P < * 0.0001) Frequency of dairy consumption: 0.39 (*P* < 0.0001) Geometric mean (±1.96SD) concentrations of Cobalamin in macrobiotic vs. control boys: 213 (107–426) vs. 484 (238–985) pmol/L Cobalamin in macrobiotic vs. control girls: 288 (112–738) vs. 458 (206–1,020) pmol/L MMA in macrobiotic vs. control boys: 0.29 (0.09–0.93) vs. 0.15 (0.06–0.43) μmol/L MMA in macrobiotic vs. control girls: 0.25 (0.09–0.70) vs. 0.17 (0.07–0.40) μmol/L Hcy in macrobiotic vs. control boys: 8.3 (5.2–13.4) vs. 7.0 (4.2–11.7) μmol/L Hcy in macrobiotic vs. control girls: 7.6 (3.8–15.1) vs. 7.2 (3.8–13.7) μmol/L	The group of macrobiotic fed children had received a macrobiotic diet until 6 y of age and had then switched to a lactovegetarian, lacto ovovegetarian, or omnivorous diet (macrobiotic adolescents). The group of macrobiotic fed children switched to a diet containing dairy products (200 g milk or yogurt and 22 g cheese/d (supplying on average 0.95 mg cobalamin/d), and fish, meat, or chicken 2–3 times/wk. In girls, meat consumption contributed more to vitamin B12 status than the consumption of dairy products, whereas in boys these food groups were equally important.	Moderate consumption of animal products after cessation of a macrobiotic diet is insufficient to restore low vitamin B12 status among adolescents
Villamor et al. (32), 2008, Colombia, Cross–sectional	*N =* 972 (49%)	8.7 [5–12]	38 item FFQ obtained by mothers to assess dietary intake among children	Vitamin B12 (pmol/L)	P for trend across quartiles of plasma B12 with: Animal protein pattern: 0.003 Cheap protein pattern: 0.75 Traditional/starch pattern: 0.45 Snacking pattern: 0.84 Adjusted differences (95%CI, P for trend) in B12 concentrations of high vs. low/no intake of: Meat: 24 (1 to 48, 0.04) Dairy: 32 (5 to 95, 0.06) Fish: 17 (−7 to 41, 0.16) Cow liver: 5 (−17 to 28, 0.08) Egg: −25 (−50 to 1, 0.12) Supplement: 9(−8 to 27, 0.31)	PCA derived patterns:Animal protein (beef/ pork/veal/lamb, chicken/turkey, milk, cheese) Cheaper protein (cow tripe/liver, spleen, chicken giblets) traditional/starch (rice, potato, plantain), snacking (candy, ice cream, packed fried snacks, soda, fruit punch). Analyses adjusted for sex, age, frequency of meat, dairy, fish, cow liver, and supplement intake.	Strong dose–dependent positive association between a pattern including frequent consumption of beef, chicken, and dairy products and plasma vitamin B12.
Hay et al. (33), 2011, Norway, Cross–sectional	*N =* 155 (44%)	2 [2–2]	7–day food records. Diary, liver pate, meat (products), fish (products).	Vitamin B12 (pmol/L) and holoTC (pmol/L)	Spearman correlations of Serum Vitamin *B1*2 with: Dairy products: 0.16 (*P < * 0.05) Serum HoloTC with: Dairy products: 0.27 (*P < * 0.01) Liver pate: 0.20 (*P < * 0.05) P for differences of geometric means by quartiles from food sources: Serum vitamin *B1*2: Dairy: *P =* 0.197 Liver pate: *P =* 0.212 Meat & meat products: *P =* 0.986 Fish & fish products: *P =* 0.865 Serum holoTC: Dairy: *P =* 0.024 Liver pate: *P =* 0.005 Meat & meat products: *P =* 0.204 Fish & fish products: *P =* 0.680	Adjusted for sex and energy intakeMMA and Hcy were not associated with animal food intake.	In this unfortified toddler population, vitamin B12 status was strongest associated with dairy intake, and with a lesser extend to liver pate'
Christian et al. (34), 2015, India, Cross–sectional	*N =* 512 (52.9%)	9.5 [9–10]	136 item Semi quantitative FFQ, including amongst others minced meat, fish, chicken, mutton, meat and fish, eggs, non–vegetarian, curd foods, milk and dairy	Vitamin B12 (pmol/L)	B (95%CI) T3 vs. T1 of intake with plasma B12: Minced meat: 0.011 (−0.077 to 0.100) Fish: 0.052 (−0.031 to 0.134) Chicken: −0.003 (−0.112 to 0.105) Mutton: 0.101 (−0.007 to 0.209) Meat & fish: 0.126 (0.041 to 0.212) Eggs: −0.031 (−0.122 to 0.061) Non–vegetarian: 0.124 (0.044 to 0.203) Curd (yogurt) foods: −0.075 (−0.157 to 0.007) Milk/dairy: −0.029 (−0.110 to 0.053)	Adjusted for age, sex, BMI, height, SLI score, maternal education, other food groups in the table except traditional fermented foods and raw vegetables, and pregnancy plasma B12 concentrations.	Meat and fish are most important animal derived B12 sources among Indian children
Manioset al. (35), 2017, Greece, Cross–sectional	*N =* 600 (51%)	11 [9–13]	Three 24 h dietary recalls (2 week days, 1 weekend day)	Hcy (μmol/L)	*B* (**p-value**) linear regression of Hcy with *B1*2 intake from: Milk: −0.120 (0.004) *B* (**p-value**) quadratic regression of Hcy with *B1*2 intake from: Milk: −0.515 (<0.001)	Adjusted for age, sex, and total vitamin B12 intake from other food sources Vitamin B12 intake from red meat and cheese were not associated with Hcy concentrations.	High vitamin B12 intake from milk was associated with lower Hcy concentrations
**PREGNANCY**							
Denissen et al. (36), 2019, The Netherlands, Cross sectional	*N =* 1,266 (100%)	32.6 ± 3.8	200 item semi–quantitative FFQ, including dairy products (28 items), meat (29 items), (shell)fish (7 items), eggs (1 item)	Vitamin B12 (pmol/L) holoTC (pmol/L) MMA (μmol/L)	%difference (95%CI) Q5 vs. Q1: Dairy–B12: 29 (21 to 37) Dairy–HoloTC: 53 (41 to 66) Dairy–MMA: −21 (−27 to −14) Meat–B12: 15 (8 to 23) Meat–HoloTC: 20 (10 to 30) Meat–MMA: −16 (−23 to −9) Fish–B12: 7 (0.5 to 13) Fish– HoloTC: 15 (7 to 24) Fish– MMA: −15 (−21 to −8) Eggs– B12: 1 (−5 to 7) Eggs– HoloTC: 9 (1 to 17) Eggs– MMA: −5 (−12 to 2)	Multivariable adjusted proportional difference in geometrical means of highest quintile relative to lowest quintile of intake. Values obtained by multiple linear regression analyses adjusted for recruitment group, age, prepregnancy BMI, education, smoking, vitamin B−12 intake from supplements, alcohol use, energy intake, vitamin B−12 intake from mixed dishes, as well as for vitamin B−12 intake from dairy, meat, fish and eggs (except for the food group of interest)	
**ADULTS (MEAN AGE POPULATION** **>18 And** **≤64 y)**							
Tuckeret al. (3), 2000, United states, Prospective	*N =* 2,999 (52%)	53.6 [26–83]	126 item Semi quantitative FFQ. Vitamin B12 intake was calculated from individual food sources. Food contributions to total vitamin B−12 was calculated, and total vitamin B12 intake was divided into vitamin B12 intake from supplements, breakfast cereals, meat, poultry, fish, dairy sources, and all other foods	vitamin B12 (pmol/L)	Prevalence of *B1*2 <185 *vs*. 148 pmol/L for: Dairy upper tertile: 13.4 vs. 6.8 Dairy middle tertile: 21.2 vs. 10.4 Dairy lowest tertile: 24.5 vs. 14.1 P difference T1 and T3 <0.001 for both cutoff levels Meat upper tertile: 17.4 vs. 8.5 Meat middle tertile: 20.4 vs. 11.4 Meat lowest tertile: 21.5 vs. 11.4 P difference T1 and T3>0.05 for both cutoff levels Odds Ratios (95%CI) of having B12 <185 or <148 pmol/L comparing each dietary pattern to high supplement intake group: Meat 57% (*n =* 740): 2.4 (1.7 to 3.4) or 2.0 (1.2 to 3.3) Milk 38%,meat 21%, fish 10% (*n =* 361): 1.6 (1.1 to 2.5) or 1.0 (0.5 to 1.8) Meat 25%, soups 24%, fish 12%, milk 10% (*n =* 592): 2.2 (1.5 to 3.3) or 1.6 (0.9 to 2.6) Fish 35%, meat 21%, other dairy 10% (*n =* 342): 2.1 (1.4 to 3.3) or 1.6 (0.9 to 2.8)	Adjusted for age and sex Adjusted for age, sex, total energy intake, total vitamin B12 intake Dietary patterns were derived from cluster analysis.	Milk appears to protect against lower vitamin B12 concentrations. Participants in all food intake groups were significantly more likely to have B12 concentrations <185 pmol/L compared to subjects in the supplement group. Only the meat group differed significantly from the supplement group in having vitamin B12 concentrations <148 pmol/L.
Gao et al. (38), 2003, China, Cross–sectional	*N =* 119 (54%)	42 [35–49]	170 item FFQ from which intake of Fruit and milk, Red meat, and refined cereals were composed	vitamin B12 (pmol/L) Hcy (μmol/L)	OR (95%CI) of having *B1*2 <221 pmol/L: Red meat vs. fruit & milk: 2.4 (0.9 to 6.3) Refined cereals vs. fruit & milk: 6.2 (1.9 to 20.8) OR (95%CI) of having Hcy>11 for women and >12 for men: Red meat vs. fruit & milk: 2.6 (0.9 to 7.4) Refined cereals vs. fruit & milk: 5.0 (1.5 to 17.5)	Adjusted for age, sex, total energy intake, BMI, smoking, alcohol use, income and education level.	The pattern high in fruits and milk was associated with a significantly lower risk of having Hcy >11 or 12 μmol/L and of having B12 <221 pmol/L compared to the pattern of refined cereals. Pattern of red meat did not differ in risk of high Hcy or low B12 compared to the fruit and milk pattern.
Koebnick et al. (40), 2005, Germany, Cross–sectional	*N =* 187 (53%)	46 [25–64]	7 day food records including 12 food groups	vitamin B12 (pmol/L) Hcy (μmol/L)	Median (P25, P75) concentrations according to type of raw food consumption: Vitamin *B1*2 concentrations: Mixed raw food: 175 (142,250) ovo–lacto–vegetarian: 143 (121,176) vegan: 126 (88,182) pmol/L Median (P25, P75) plasma Hcy concentrations: Mixed raw food: 14.7 (11.9,18.3) ovo–lacto–vegetarian: 17.1 (13.1,20.2) Vegan: 18.5 (13.5,28.9) μmol/L	Unadjusted analyses Mixed raw food diet included raw meat and fish	Individuals who consumed a mixed raw food diet had highest vitamin B12 and lowest Hcy concentrations whereas those consuming a stricht vegan diet had lowest vitamin B12 and highest Hcy concentrations.
Hao et al. (42), 2007, China, Cross–sectional	*N =* 2,407 (51%)	49 [35–64]	Semi quantitative FFQ. Animal–based foods were classified in dairy, egg, animal meat and fish.	vitamin B_12(_pmol/L)	Adjusted OR (95%CI, P for trend) for having vitamin B12 deficiency (<185 pmol/L): T3 vs. T1 dairy: 0.5 (0.4 to 0.7, <0.001) Q5 vs. Q1 egg: 0.8 (0.6 to 1.1, 0.196) Q5 vs. Q1 meat: 1.0 (0.7 to 1.4, 0.163) Q5 vs. Q1 fish: 0.4 (0.3 to 0.5, <0.001)	Intake food source divided in tertiles (dairy) or quintiles (egg, animal meat, fish) Adjusted for region, area (urban, rural), gender, age, season	Higher consumption of dairy and fish was associated with a lower likelyhood of having B12 concentrations <185 pmol/L compared to low or intermediate consumption.
Vogiatzoglou et al. (43), 2009, Norway, Cross–sectional	*N =* 3,067 (55%)	[47–49]	169 item FFQ	Vitamin B12 (pmol/L)	Adjusted mean (95%CI) plasma vitamin B12 concentrations in Q4 vs. Q1 B12 intake from: dairy: 385 (376 to 395) vs. 323 (314 to 332) milk: 388 (379 to 397) vs. 331 (324 to 338) Cheese: 370 (362 to 378) vs. 346 (338 to 335) Meat: 362 (354 to 369) vs. 355 (344 to 366) (shell) fish: 375 (366 to 385) vs. 339 (331 to 346) Eggs: 358 (349 to 366) vs. 356 (350 to 363) P for trend <0.001, except for meat (*P* = 0.189) and eggs (*P* = 0.837)	Adjusted for sex, energy, use of B vitamin containing supplements, total intake of other food groups	Dairy and fish are significant contributors to plasma vitamin B12. Vitamin B12 appears to be more bioavailable from dairy products than from other animal products.
Yakub et al. (44), 2010, Pakistan, Cross–sectional	*N =* 872 (59%)	32.4 [18–60]	15 item food group frequency questionnaire composing dietary patterns	Vitamin B12 (pmol/L) Hcy (μmol/L)	Adjusted mean plasma vitamin B12 concentrations in Q4 vs. Q1 (P diff) intake from: Prudent diet: 322 vs. 317 (P diff = 0.85) High animal protein diet: 335 vs. 312 (P diff = 0.56) High plant protein diet: 325 vs. 326 (P diff = 0.80) Adjusted mean plasma Hcy in Q4 vs. Q1 (P diff) intake from: Prudent diet: 13.97 vs. 15.78 (P diff = 0.26) High animal protein diet: 18.58 vs. 13.29 (P diff <0.001) High plant protein diet: 12.50 vs. 18.40 (P diff <0.001)	Adjusted for age and sex Patterns identified by factor analyses: Prudent pattern: high intake of eggs, fish, uncooked vegetables, juices, and bananas and other fruits. High animal–protein pattern: high intake of meat, chicken, wheat, bananas, and tea with milk. High plant–protein pattern: high intake of cooked vegetables and legumes and a small intake of meat	Patterns high in animal and plant proteins were associated with lower Hcy, but not with vitamin B12 concentrations.
Murakami et al. (45), 2013, Japan, Cross–sectional	*N =* 1,050 (100%)	20 [18–22]	Diet history questionnaire including 17 foods	Hcy (μmol/L)	Adjusted geometric mean (95%CI) Hcy concentrations were significantly lower in Q5 vs. Q1 dairy intake (P for trend 0.02). Hcy did not differ across quintiles of (shell)fish, meats, and egg consumption	Adjusted for survey year, region, municipality level, current smoking, current alcohol drinking, supplement use, physical activity, BMI, energy intake, intakes of other foods.	High consumption of dairy products was associated with lower Hcy concentrations
**ELDERLY (MEAN AGE POPULATION** **>002065 y)**							
Kwan et al. (46), 2002, Puerto Rica and Dominicans, Cross–sectional	*N =* 603 (58%) (Hispanic (*n =* 449), non–Hispanic white (*n =* 154))	76.5 [60–93]	Semi qualitative FFQ. Total vitamin B12 intake divided into vitamin B12 intake from supplements, breakfast cereals, dairy sources, eggs, meat, poultry, fish, and all other foods	Vitamin B12 (pmol/L)	Proportions of B12 <185 pmol/L of vitamin B12 intake from: Hispanics: Dairy: 15.8 (T1), 14.2 (T2), 23.6 (T3), P for diff>0.05 Meat: 15.5 (T1), 15.6 (T2), 22.0 (T3), P for diff>0.05 *Non*–Hispanics whites: Dairy: 9.4 (T1), 10.5 (T2), 20.0 (T3), P for diff>0.05 Meat: 9.8 (T1), 20.6 (T2), 10.0 (T3), P for diff>0.05	Adjusted for age, sex, and energy intake.	Dairy and meat consumption are not significantly related to vitamin B12 status
Lasheras et al. (47), 2003, Spain, Cross–sectional	*N =* 140 (58%)	Men: 73.3 women: 74.2 [60–80]	FFQ for dietary intake, individual foods vegetables, legumes, fruit, cereals, potatoes, fish, meat, eggs, milk and dairy, other foods.	Hcy (μmol/L)	Multiple linear regression with beta (95%CI) for Hcy and intake of: Meat: −0.083 (−0.035 to 0.010) Milk and dairy products: 0.004 (−0.020 to 0.021) Total dietary score: −0.156 (−0.545 to −0.015)	Adjusted for sex, age, and serum creatinine Total diet score based on quartiles of the intakes (grams per day) of main food groups contributing to intake of B–vitamins: i.e., meat, fish, milk, dairy, fruit, and vegetables.	Only the dietary pattern characterized by high intakes of B vitamin–rich foods was associated with lower Hcy concentrations and lower proportion of high Hcy.
Ledikwe et al. (48), 2004, United states, Cross–sectional	*N =* 179 (55%)	76.5 [66–80]	24 h recall, 2 months interval during 10 months, categorized 6 main food sources; High nutrient dense; vegetables, fruit, milk, poultry fish. Low nutrient dense; dairy desserts, meat	Vitamin B12 (pg/mL)	Least Square Mean (95%CI) vitamin B12 (pg/mL): Low–nutrient dense pattern: 455 (406–504) High–nutrient dense pattern: 556 (493–618) P for difference 0.03 Least Square Mean (95%CI) Hcy (μmol/L): Low–nutrient dense pattern: 9.9 (9.1–10.7) High–nutrient dense pattern: 9.9 (8.9–10.8) P for difference 0.981 OR (95%CI) of having B12 <350 pg/mL while consuming low nutrient dense pattern group compared to high nutrient dense pattern: 2.15 (0.93–4.96)	Significance tests adjusted for energy intake, age, sex, tobacco use, alcohol use Low–nutrient–dense pattern: higher intake of breads, sweet breads/desserts, dairy desserts, processed meats, eggs, and fats/oils High–nutrient– dense pattern: higher intake of cereals, dark green/yellow vegetables, other vegetables, citrus/ melons/berries, fruit juices, other fruits, milks, poultry, fish, and beans	Consumption of a high–nutrient–dense dietary pattern was associated with higher vitamin B12 concentrations compared to a low nutrient dense dietary pattern. Hcy concentrations did not differ between high and low nutrient dense diets.
Vogiatzoglou et al. (43), 2009, Norway, Cross–sectional	*N =* 2,861	[71–74]	169 item FFQ	Vitamin B12 (pmol/L)	Adjusted mean (95%CI) plasma vitamin B12 concentrations in Q4 vs. Q1 B12 intake from: Dairy: 358 (348,368) vs. 318 (309, 328) Milk: 357 (347,367) vs. 317 (307, 327) Cheese: 343 (332,355) vs. 337 (328, 347) meat & meat products: 342 (332, 354) vs. 340 (332, 349) Fish and shellfish: 359 (350, 369) vs. 321 (311, 330) eggs: 334 (324, 344) vs. 339 (331,347) P for trend <0.01, except for cheese (*P* = 0.707), meat (*P* = 0.522), and eggs (*P* = 0.677)	Adjusted for sex, energy, use of B vitamin containing supplements, total intake of other food groups	Dairy and fish are significant contributors to plasma vitamin B12. Vitamin B12 appears to be more bioavailable from dairy products than from other animal products.
Brouwer– Brolsma et al. (49), 2015, The Netherlands, Cross–sectional	*N =* 600 (42%)	72 [>65]	190 item FFQ including meat, fish and shell fish, eggs and dairy products.	Vitamin B12 (pmol/L)	Probability (95%CI) of having serum B12 > 200 pmol/L (T3 vs. T1). B12 intake from: Total vitamin B12 intake: 1.20 (1.06, 1.35) Meat: 1.22 (1.08, 1.37) (Shell)fish: 1.16 (1.04, 1.30) Eggs: 1.05 (0.93, 1.18) Dairy: 1.24 (1.10, 1.39)	Adjusted for age, sex, BMI, education, alcohol intake, physical activity, smoking, creatinine, total energy intake, intake of other vitamin B_12_ containing food items.	Higher intakes of dairy, meat, and fish and shellfish were significantly associated with higher vitamin B12 concentrations, with meat and dairy (predominantly milk were the most potent sources)

### Infants and Children

Two case-control studies among infants (29) and children (30) investigated the effects of a macrobiotic dietary regime (no animal foods) on vitamin B12 biomarkers. Plasma vitamin B12 concentrations were significantly lower among macro-biotic fed infants (*n* = 47) as compared to their omnivorous fed controls (*n* = 56) (29). In another study, adolescents who had received a macrobiotic diet until 6 y of age and had then switched a diet containing animal products (*n* = 73) still had significantly lower vitamin B12 concentrations and higher concentrations MMA, but comparable Hcy concentrations, as compared to their age-matched controls who consumed an omnivorous diet from birth onwards. These results suggest that switching from a macrobiotic diet to moderate consumption of animal food products is inadequate to restore vitamin B12 status among children with a low vitamin B12 in early childhood (30). A Swedish study among adolescents [mean (SD) age: 17.5 (1.0) year] compared vitamin B12 intake between 30 vegans (15 males and 15 females) and 30 sex-, age-, and height-matched omnivores. This study revealed significant differences in vitamin B12 intake between vegans and omnivores, with vitamin B12 intakes of 0.0 and 0.1 μg/day for vegan females and males, respectively, and intakes of 5.0 and 5.9 μg/day for omnivorous females and males, respectively (P for differences < 0.001)(50).

In contrast to following a well-defined dietary regime, a Colombian study identified 4 dietary patterns derived from an 28-item FFQ based on principal component analysis. Patterns included diets rich in (1) animal protein (e.g., beef/pork/veal/lamb, chicken/turkey, milk, cheese), (2) cheaper protein (e.g., cow tripe/liver, spleen, chicken giblets), (3) traditional/starch (e.g., rice, potato, plantain), and (4) snacking products (e.g., candy, ice cream, packed fried snacks, soda, fruit punch). Only the pattern rich in animal protein was significantly positively associated with plasma vitamin B12 (P for trend = 0.003). This study also studied individual animal food groups, and fully adjusted differences in plasma vitamin B12 for low vs. high consumers were significant only for meat, but not for dairy, fish, cows liver, and eggs (32). In line with these findings, a study conducted in India (*n* = 512) also showed statistically significant positive associations of the meat and fish group with plasma vitamin B12 in fully adjusted models, but not for other animal products (34). Others observed inverse associations between vitamin B12 intake from milk with plasma Hcy, but not for vitamin B12 intake from red meat or cheese (35). One study measured multiple biomarkers for vitamin B12 status. Serum MMA and Hcy concentrations were not correlated with animal food groups, whereas correlation coefficients of serum vitamin B12 and holoTC with dairy intake were 0.16 (*P* < 0.05) and 0.27 (*P* < 0.01), respectively. In addition, intake of liver pate correlated with holoTC (*r* = 0.20, *P* < 0.05). None of the vitamin B12 biomarkers were associated with fish or eggs intakes (33).

When considering different animal products within individual studies among children, differences in vitamin B12 concentrations were most pronounced when comparing high vs. low intake of dairy products, followed by meat and fish intake (32), and dairy products showed stronger correlations with vitamin B12 and holoTC concentrations compared to liver pate, meat and fish (33). In another study, only a combined group of meat and fish was associated with vitamin B12 concentrations, whereas the individual components fish, chicken, eggs, and dairy were not related to plasma vitamin B12 (34).

### Pregnancy

Only one study among pregnant women (*n* = 1266) was identified that addressed the association of vitamin B12 intake from dairy, meat, (shell)fish, and eggs with circulating levels of vitamin B12 biomarkers, and presence of vitamin B12 deficiency in week 34–36 of pregnancy. Results showed that vitamin B12 from dairy, meat and fish, but not eggs, independently contributed to plasma concentrations of total vitamin B12, holoTC and MMA, as shown by statistically significant dose-response relationships. Vitamin B12 intake from each of these products groups was also independently associated with a reduced odds of vitamin B12 deficiency (holoTC <35 pmol/L and MMA >0.45 μmol/L). Egg-derived vitamin B12 was negatively associated with holoTC but not associated with other vitamin B12 biomarkers (36).

### Adults

Those studies addressing specific animal products revealed that high dairy consumption was associated with significantly lower prevalences of vitamin B12 concentrations <185 pmol/L (3, 42) and 148 pmol/L (3) compared to low dairy consumption(3), significantly higher vitamin B12 concentrations among high milk and cheese consumers compared to low consumers (43), and significantly lower Hcy concentrations with high dairy intake compared to low dairy intake (45). Similarly, those with a fish consumption in the highest quintile had a significantly lower odds of having vitamin B12 deficiency compared to adults who had a fish consumption in the lowest quintile (42), and plasma vitamin B12 concentrations were significantly higher in those consuming high amounts (fourth quartile) compared to low fish consumers (first quartile) (43). In contrast, analyses on meat consumption did not show any relation of meat consumption with vitamin B12 deficiency (3, 42). Moreover, plasma vitamin B12 concentrations (43) and serum Hcy (45) did not differ between high and low meat consumers. Egg consumption was also not related to plasma vitamin B12 status (42, 43, 45). None of the studies investigated the link between animal food products with MMA or holoTC concentrations in adults.

A number of studies described vitamin B12 intake or vitamin B12 biomarkers among omnivores, vegetarians and vegans. All studies consistently observed that vitamin intake was lowest, intermediate and highest among vegans, vegetarians and meat-eaters, respectively (51–55). Similarly, studies also observed lowest, intermediate and highest vitamin B12 concentrations among vegans, vegetarians and meat-eaters, respectively (51, 54–56), or with holoTC concentrations(51). In line with this, prevalences of vitamin B12 deficiency were highest among vegans and lowest among omnivorous (52, 54, 56), although it should be noted that these studies used different criteria to define vitamin B12 deficiency. Other studies addressing dietary patterns in relation to vitamin B12 status have used different approaches to define patterns. Tucker et al derived patterns by cluster analysis. Food groups that contributed to vitamin B12 were entered into the analysis as percentages of total individual vitamin B12 intake. The cluster procedure assigns individuals to predetermined numbers of clusters in a manner that maximizes the difference across groups for the included variables. Factor analyses reveal that 6 patterns led to the clearest separation of vitamin B12 sources, being (1) supplements (61% Supplements, 11% meat), (2) meat (57% meat), (3) milk (38% milk, 21% meat, 10% fish), (4) cereal (37% Cereal, 17% meat, 12% milk, (5) meat and soups (25% Meat, 24% soups, 12% fish, 10% milk), and (6) fish (35% Fish, 21% meat, 10% other dairy). Plasma vitamin B12 concentrations were significantly lower in the meat pattern than in the cereal and milk patterns, despite similar average vitamin B12 intakes in these 3 groups. Subjects in all food intake groups were significantly more likely to have plasma vitamin B-12 concentrations <185 pmol/L compared to subjects in the supplement group, with odds ratios ranging from 1.6 for the milk group to 2.4 for the meat group. For the likelihood of plasma vitamin B-12 concentrations <148 pmol/L, the meat group was the only group that differed significantly from the supplement group [OR (95%CI) = 2.0 (1.2–3.3)] (3). Another study also used factor analyses to identify major dietary patterns. Three patterns were defined as (1) prudent diet (high intake of eggs, fish, uncooked vegetables, juices, bananas, and other fruits), (2) high animal-protein diet (high intake of meat, chicken, wheat, bananas, and tea with milk), and (3) high plant-protein diet (large intake of cooked vegetables and legumes and a small intake of meat). High intakes of the prudent dietary pattern and the plant protein dietary pattern (quartile 4) compared with lowest intake (quartile 1) were associated with a reduced odds of hyperhomocysteinemia (Hcy > 15 μmol/L), with OR (95% CI) of 0.52 (0.30–0.90) and 0.42 (0.25–0.69), respectively. In contrast, a high consumption of the animal-protein diet was positively associated with hyperhomocysteinemia [OR (95% CI) quartile 4 vs. quartile 1 = 2.10 (1.22–3.60). Vitamin B12 concentrations did not differ across quartiles of any of the diets (44). Finally, another study investigated if vitamin B12 and Hcy concentrations differed across different degrees of vegetariarism (vegan, ovo-lacto-vegetarian, and mixed raw food diet including raw meat and fish). This study revealed that consumption of a vegan diet had lowest median vitamin B12 concentrations and highest Hcy concentrations and consumption of a pattern with mixed raw foods had highest vitamin B12 and lowest Hcy concentrations (40).

### Elderly

Five observational studies were identified among elderly (average age population >65 y), out of which 4 focussed on specific animal products (43, 46, 47, 49) and two on dietary patterns (47, 48). A study investigating vitamin B12 intake from supplements, breakfast cereals, dairy and meat consumption revealed that only cereal, but not dairy or meat consumption was related to vitamin B12 concentrations and proportions of B12 concentrations <185 pmol/L. In line with this, meat and milk and dairy products were not associated with Hcy concentrations (47). However, other studies showed that high consumption of dairy and fish were accompanied by higher plasma vitamin B12 concentrations compared to low consumption of these food groups(43), and that high intakes (T3 vs. T1) of meat, (shell)fish, and dairy were associated with an increased odds of having vitamin B12 concentrations >200 pmol/L (49).

A dietary pattern characterized by high intakes of foods rich in B-vitamins, such as meat, fish, milk, dairy, fruit, and vegetables, was associated with lower mean Hcy concentrations (47). Another study comparing consumption of high vs. low nutrient-dense dietary pattern revealed higher vitamin B12 concentrations in those consuming a high nutrient dense pattern compared to those consuming a low nutrient dense dietary pattern. Hcy concentrations did not differ between these the high and low nutrient dense patterns. In this study, a high nutrient-dense pattern was defined as higher intake of cereals, dark green/yellow vegetables, other vegetables, citrus/ melons/berries, fruit juices, other fruits, milks, poultry, fish, and beans, whereas a low-nutrient-dense pattern consisted of higher intake of breads, sweet breads/desserts, dairy desserts, processed meats, eggs, and fats/oils (48).

### Summary of General Findings and Considerations

Dairy consumption seems to be the strongest determinant of vitamin B12 concentrations. However, when comparing the magnitude of the relation of dairy, meat, fish or egg consumption with vitamin B12 status it is essential to adjust statistical analyses for vitamin B12 intake from other animal food products, which was done in 5 studies (3, 34, 35, 43, 49). In addition, the specific individual food items representing dairy, meat, and fish consumption could not always be derived from the individual studies, which hampers direct comparison between studies. Finally, nutrient-density of different dairy (milk, yogurt, cheese, curd cheese), meat (chicken, pork, veal), and fish (lean vs. fatty) differs considerably. There is a knowledge gap regarding the bioavailability of vitamin B12 from these different animal food products. In addition, associations of different animal product groups with MMA and holoTC remain largely unknown.

## Vitamin B12 Intake or Status and Health Outcomes

This section addresses the associations between low vitamin B12 intake or status (defined by abnormal biomarkers) and several health outcomes from epidemiological studies performed in vulnerable population groups.

### Maternal Vitamin B12 Status and Pregnancy

Vitamin B12 deficiency can cause megaloblastic anemia/pernicious anemia (57). Women with untreated pernicious anemia have often infertility problems or repeated abortions. When women were diagnosed with vitamin B12 deficiency and had received vitamin B12, pregnancy occurred (58–60).

The metabolisms of vitamin B12 and folate interact. Supplementation of folic acid before pregnancy and in the first pregnancy trimester reduces the risk of neural tube defects (NTDs) in the child. An inverse association has been reported between NTDs risk and vitamin B12 status or polymorphisms in vitamin B12 metabolizing enzymes (61–64). Ray et al. have shown that a low serum holotranscobalamin (<55.3 pmol/L) at 15–22 weeks of gestation was associated with the risk of NTDs in a study that was done in Ontario after the fortification with folic acid (65). There is a general agreement that vitamin B12 concentrations >250 pmol/L in women entering pregnancy is associated with low risk for NTDs compared to when vitamin B12 is below this level (63). These findings may suggest that supplementation with vitamin B12 may reduce the risk for NTD. However, it is unknown if supplementation with both folic acid and vitamin B12 decreases the number of births with a NTD compared to supplementation with folic acid alone.

Maternal vitamin B12 status determines vitamin B12 status of the child at birth and thereafter. Vitamin B12 in neonates at birth is higher than that in plasma of the mother, but it generally declines in the infants after birth. In a nested case-control study, concentrations of vitamin B12, Hcy, and MMA were assessed in healthy pregnant women (*n* = 114) from week 18 of pregnancy through 6 mo postpartum and related to infant cobalamin status at 6 mo, and compared with healthy, never-pregnant women aged 18–40 y controls (*n* = 123). Compared to controls, vitamin B12, Hcy and MMA were lower in pregnant women at 18 w of pregnancy. Vitamin B12 significantly decreased from week 18 to week 36 of pregnancy and increased again by 6 wk postpartum, whereas Hcy and MMA concentrations increased from week 18 of pregnancy to 6 wk postpartum. Infant vitamin B12 concentration at 6 months correlated with maternal vitamin B12 concentration during pregnancy and postpartum (rho = 0.36–0.55, *P* < 0.001). A maternal vitamin B12 concentration <394 pmol/L during week 18 of pregnancy was associated with an increased risk (OR: 4.2; 95% CI: 1.5, 11.5) of infant vitamin B12 deficiency at 6 mo (defined as tHcy ≥6.5 μmol/L) (66). Breastfed infants are at risk for deficiency in this period if their depleted mothers are not taking vitamin B12-containing supplements (67). Most cases of infantile vitamin B12 deficiency become manifested between 6 and 11 months of age. Neuromuscular and growth or developmental disorders or cerebral atrophy can occur. Symptoms such as irritation, feeding difficulties, stunting, or anemia have been reported in deficient neonates. Vitamin B12 deficiency may leave residual neurological abnormalities (68).

### Breastfeeding

Prolonged breastfeeding is related to food insecurity and represent a problem in many parts of the world where the mothers have multiple micronutrient deficiencies. In Indian children (mean age 16 months) from families of low to middle socioeconomic status, prolonged breastfeeding was associated with stunting, anemia, low weight, or wasting (low weight-for-length) in the child (69, 70). A causal role for vitamin B12 deficiency in childhood stunting is possible, but not well-investigated due to possible confounding by intestinal infections, protein deficient diet or multiple nutrient deficiencies that can affect stunting (71–73). Maternal and/or infant vitamin B12 status has been related to infant physical growth (74), anemia, and cognitive and mental function (75). However, it is unknown if requirements for vitamin B12 in pregnant and lactating women should be increased, and if improving maternal or child vitamin B12 status can improve the outcome such as anemia (76) and cognitive development in the child. There are currently some studies ongoing on this topic (77).

### Elderly

Several epidemiological studies reported associations between vitamin B12 biomarkers and brain health, cognitive function, or bone health [reviewed in (78)]. In addition, some evidence appears to suggest that lower B12 status is related to increased pro-oxidant and decreased antioxidant status (79). In a 5-y follow up study among dementia-free elderly people, vitamin B12 in the lowest tertile (<308 pmol/L) or holoTC (< 54 pmol/L) was negatively associations with accelerated brain volume loss, compared with those with higher levels (80). No such association was observed for MMA and Hcy with brain volume loss. In contrast, Smith et al., observed an association between high plasma Hcy and MMA and the risk of cognitive impairment in elderly people who were free of dementia at baseline, while higher holoTC and vitamin B12 showed a negative association with the risk of cognitive impairment (81). Most intervention studies to lower tHy have used multivitamins containing folic acid and vitamin B12 among other vitamins, and studies have shown a protective effect of multivitamins containing vitamin B12 on global cognition (82), brain shrinkage (83), or quality of life scores (84).

In a 6-y follow up study in Swedish elderly men (*n* = 790; age range 70–81 years), lowered holoTC was associated with an increased risk of fracture (hazard risk for the lowest tertile of holoTC = 1.74; 95% CI 1.12–2.69) (85), while Hcy and MMA were not associated with bone mineral density or fracture risk. In a nationally representative cross-sectional study in U.S. women ≥50 years, Baily et al., have reported an association between elevated Hcy and MMA and the risk of lumbar spine osteoporosis, but B12 and MMA were not associated with bone mineral density (86). In an update meta-analysis including RCTs on the association between homocysteine-lowering trials and fracture risk in elderly people (87)., Garcia Lopez et al., found no association between lowering Hcy (using folic acid and vitamin B12) and the risk of fractures (87).

In general, the evidence from homocysteine-lowering trails by B-vitamins on cognition or bone fracture is mixed and there are several negative studies (78). There is some evidence that vitamin B12 supplementation could have positive effects on health in elderly people who are vitamin B12-deficient. Nevertheless, more research in this group of elderly people is still warranted.

### Special Considerations

#### The Health Significance of High Folate and Low Vitamin B12

There is some concern about supplementing high doses of folic acid to women of reproductive age with low vitamin B12 intake. Supplementing folic acid >1 mg/d is common in many parts of the world where low vitamin B12 is endemic, thus causing unbalanced intake and status of the two B-vitamins. Vitamin B12 deficiency is common in pregnant women from many countries such as Colombia (88), Brazil (89), or India (90). Using multivitamin supplements before pregnancy is not common and is related to education and income level (91). Imbalanced levels of folate and vitamin B12 (i.e., high folate and low B12) have raised some concern, although this topic is not well-investigated yet.

Due to low animal source foods, Indian women are a good example of a population with imbalanced folate-to-B12 ratio. A small observational study including Indian women at 36 weeks of gestation and their newborn children within 24 h after birth reported a negative association between folate-to-B12 ratio and birth weight, birth length, and head and chest circumferences (92). It has been reported that neonates born to Indian women with low B12 intake, consuming >1 mg/d of folic acid, and those with a low B12-to-total folate intake ratio are at increased risk of being born small for gestational age (93). Moreover, high red blood cell (RBC)-folate in Indian pregnant women has been related to adiposity in their children (94) and to increased risk of insulin resistance in the children if maternal plasma B12 was also low (94, 95). The risk of gestational diabetes was higher in vitamin B12-deficient Indian women (95), and the risk of persistent diabetes in the deficient women with gestational diabetes was higher in those women with higher folate status (95).

In UK pregnant women, both folate and vitamin B12 status showed inverse associations with maternal BMI (96). Vitamin B12 insufficiency was also associated with insulin resistance in those women (96). Women with a combination of low plasma vitamin B12 (<170 pmol/L) and folate (<10.3 nmol/L) had the highest BMI, while those with high vitamin B12 (>238 pmol/L) and folate status (>18.3 nmol/L) had the lowest BMI (96). Another study in UK pregnant women (beginning of the 3rd trimester) observed a negative association between maternal vitamin B12 and the risk of obesity and gestational diabetes (97). In pregnant women with gestational diabetes, the risk for fetal macrosomia was higher in the highest folate quartile and lowest vitamin B12 quartile (97). In a study among Spanish women, maternal folate was negatively associated with insulin sensitivity (HOMA-IR test), while low vitamin B12 was associated with insulin resistance (98). Given these close interactions between vitamin B12 and folate, it is not unlikely that other nutrient-nutrient interactions occur, for example with n-3 fatty acids. As such, future studies may also take nutrient-density of specific animal food products or dietary patterns into account.

#### The Health Significance of High Vitamin B12 Intake and Status

High dietary intake of vitamin B12 has not been shown to be disadvantageous. Supplemental forms of vitamin B12 are considered safe and there is no evidence-based Tolerable Upper Intake Level for vitamin B12. However, elevated plasma concentrations of vitamin B12 (often defined as plasma vitamin B12 levels >600, >800, or >1,000 pmol/L) in individuals not receiving supplemental vitamin B12 have been described in studies in patients with different cancers, liver diseases, or type 2 diabetes (99) that were later attributed to renal dysfunction (100, 101). The clearance of a single dose of radiolabeled vitamin B12 has been shown to be delayed in patients with renal dysfunction (101). Studies conducted in hospital settings have shown that elevated plasma vitamin B12 is associated with elevated plasma levels of liver enzymes and creatinine or albuminurea (102) and several clinical conditions such as chronic kidney disease, diabetes, liver disorders (of any etiology), alcoholism, or malignancies (100, 103, 104).

Elevated plasma vitamin B12 has been shown to predict future cancer (105), cardiovascular mortality in cohort studies (106), and levels above 400 pmol/L may predict short term mortality (within 90 days) irrespective of the cause of death in hospitalized elderly patients (107). A prospective study on 161 patients with different cancers investigated serum vitamin B12 concentrations and the time of death (108). The global median survival time was 45 days (CI 95%: 32–56 days). The highest mortality corresponded to the highest vitamin B12 levels. A significant link was found between elevated vitamin B12 (>600 pmol/l) and the presence of metastasis, a tumor or liver problems (108). In a recent study based on primary care database, the incidence rate ratio for cancer was 4.72 (95% confidence interval: 3.99–5.58) in persons with vitamin B12 >1,000 pmol/L compared to those with a low vitamin B12 levels after multivariate adjustments (109).

Notably, a causal role for vitamin B12 in future diseases or mortality cannot be assumed based on the presence of elevated plasma vitamin B12 levels. High vitamin B12 test results could be due to supplementation (i.e., long storage time), release from damaged tissues, or reduced kidney excretion. In all instances high plasma vitamin B12 levels are likely to be too unspecific to be used as a screening test for existing tumors or to predict future health outcomes. The likelihood of detecting cancer in patients with high vitamin B12 test has not been studied. Considering the high rate of false positive results (with seriously negative impact on patients), there is currently no evidence to initiation of further cancer diagnostic tests in subjects with plasma vitamin B12 levels >600 pmol/L.

### Consideration

Studies reporting on the relationship between vitamin B12 intake and health outcomes have limitations due to methodological variations related to quantifying the intake, differences in population characteristics and to the fact that clinical outcomes such as anemia or neuropathy are late manifestations of the deficiency and are not specific for vitamin B12 deficiency.

## Overall Summary

The current recommendation is to decrease consumption of animal foods and increase consumption of plant foods, as recently suggested by the EAT-Lancet commission (110). However, a major concern of diets low or without animal products is the risk of vitamin B12 deficiency. This review showed that a total intake of vitamin B12 from the diet between 4 and 7 μg/d is associated with normal plasma vitamin B12 and MMA and thus appears to be adequate to maintain body vitamin B12 status in adults. However, this intake might not be sufficient if people have difficulties in chewing foods, releasing the vitamin from its food binding, and/or absorbing it due to intrinsic factor antibodies or medications (25, 26). It is currently unknown if vitamin B12 requirements should be age-specific, since vitamin B12 deficiency is common in elderly.

When considering specific animal food products, dairy consumption seemed to be a stronger determinant of vitamin B12 concentrations than meat, fish and eggs. However, nutritional composition of different dairy (milk, yogurt, cheese, curd cheese), meat (chicken, pork, veal), and fish (lean vs. fatty) differs considerably, and bioavailability of vitamin B12 from these different animal food products together with potential interactions between vitamin B12 and other nutrients from these nutrient-dense animal products are unclear. Therefore, nutrient-density or well-known interactions between nutrients, such as folate and vitamin B12, should also be considered when studying the relations of intake on status or health.

## Author Contributions

SE, RO, SH, and MV substantial contributions to the conception or design of the work. All authors drafting the work or revising it critically for important intellectual content. All authors provide approval for publication of the content. All authors agree to be accountable for all aspects of the work in ensuring that questions related to the accuracy or integrity of any part of the work are appropriately investigated and resolved. SE, RO, SH, and MV selected extracted relevant papers of this manuscript. SE, RO, and SH wrote the manuscript. SE had primary responsibility for final content. All authors read and approved the final manuscript.

### Conflict of Interest Statement

EvdH was employed by company FrieslandCampina. The remaining authors declare that the research was conducted in the absence of any commercial or financial relationships that could be construed as a potential conflict of interest.

## Footnotes

^1^https://wateetnederland.nl/resultaten/vitamines-en-mineralen/bronnen

## Abbreviations

IF: intrinsic factor
holoTC: holotranscobalamin
MMA: methylmalonic acid
Hcy: total homocysteine
NTD: neural tube defects.
